# Exploring the mental health research priorities of parents with depression and their children

**DOI:** 10.1136/bmjment-2024-301279

**Published:** 2025-06-22

**Authors:** Victoria Powell, Emma Meilak, Chloe Booth, Lucy Owen, Lucy Brookes-Howell, Anita Thapar, Frances Rice

**Affiliations:** 1 Wolfson Centre for Young People’s Mental Health, Cardiff University, Cardiff, Wales, UK; 2 Centre for Trials Research, Cardiff University, Cardiff, Wales, UK

**Keywords:** Depression, Child & adolescent psychiatry, Depression & mood disorders, Adult psychiatry

## Abstract

**Background:**

While patient and public involvement (PPI) in research is growing, PPI in the setting of research priorities at an early stage of the research process has been limited to date. Where research priorities have been assessed, this has been done by working with members of the general public. Research priorities are likely to vary between different groups, and families affected by depression have been recognised as an important group for research.

**Objective:**

We aimed to explore the mental health research priorities of parents with a history of depression and their children.

**Methods:**

Data came from the Early Prediction of Adolescent Depression (EPAD) study—a UK longitudinal cohort study of parents with a history of depression and their children. During interviews, parents (n=161) and their young adult children (n=131) were asked open-ended questions about their research priorities. Responses were analysed using qualitative content analysis.

**Findings:**

Parents and their young adult children highlighted the following research priority categories: treatment and intervention, including prevention and early intervention, public understanding of mental health, environmental or social factors that might contribute to poor mental health, the role of genetics in intergenerational transmission, and a developmental and intergenerational approach to research.

**Conclusions:**

While prior research has identified the importance of intervention and social factors, our study also identified public understanding of mental health and aetiological research, particularly on the contribution of genetics relative to environmental factors, as priorities for parents with depression and their children.

**Clinical implications:**

Findings highlight the value of involving diverse groups in priority-setting exercises, including groups that are recognised as important for research, to allow their views to be incorporated into agenda-setting initiatives, including for research funding.

**WHAT IS ALREADY KNOWN ON THIS TOPIC**

- While there has been growing interest and investment in patient and public involvement (PPI) in research, PPI in the setting of mental health research priorities has been limited, and key exceptions to this have been conducted with members of the general public. Families affected by depression have been identified as an important research group, but their views on priorities for mental health research are not clear.

**WHAT THIS STUDY ADDS**

- This study used qualitative content analysis to explore the research priorities of parents with a history of depression and their young adult children and identified the following priorities: treatment and intervention, including prevention and early intervention, public understanding of mental health, environmental or social factors that might contribute to poor mental health, the role of genetics in intergenerational transmission, and a developmental and intergenerational approach to research.

**HOW THIS STUDY MIGHT AFFECT RESEARCH, PRACTICE OR POLICY**

- These findings have implications for future mental health research directions and illustrate the value of including diverse groups in priority-setting exercises because they bring a distinct perspective.

## Background

 There is increasing appreciation internationally that patient and public involvement (PPI) is important in all stages of the research process.^1^ A growing number of research funders have now begun to mandate PPI, and scientists are increasingly including the voices of those with lived experience of mental health difficulties in mental health research.^2^

However, PPI in setting mental health research *priorities* in the early stages of the research process, informing the topics that research should focus on, has been limited to date.^2 3^ Some notable exceptions are surveys completed by members of the public or stakeholder workshops.^4^,^7^ However, these have focused largely on identifying priorities for youth mental health interventions and service provision as opposed to identifying priorities for mental health research questions.

It seems highly likely that research priorities will differ across groups, and it is essential to ask groups who have been identified as an important focus for research about their views on research priorities. One particular group of interest is parents with depression and their children. This group has been identified as a priority group for research^8^ because depression is a leading cause of global disability and intergenerational transmission of mental health disorders and other poor outcomes is common in children with depressed parents.^9^ Relevant research that has included the views of individuals who have experienced depression has addressed questions such as the outcomes that matter to them in treatment studies^10^ and understanding how depression is experienced by young people.^11^ However, we are not aware of a study that focuses on understanding the mental health research priorities of parents with depression and their children, despite this being an important group to involve in research priority-setting exercises. Such information could be useful in informing research funding priorities.^12^ Increasing the diversity of stakeholders who are consulted is paramount to ensuring greater inclusivity in priority-setting exercises^2^ and that research is focused on areas that are important to the people that the research affects.^13 14^

### Objective

The current study aimed to explore the research priorities of parents and their young adult children from a UK cohort study of parents with a history of major depressive disorder (MDD) and their children. We used qualitative content analysis (QCA) of data from face-to-face interviews.

## Methods

### Sample

Data came from the Early Prediction of Adolescent Depression (EPAD) study—a UK, prospective, longitudinal cohort study of parents with a history of recurrent MDD and their children.^15^ At baseline, 337 parent-child dyads, including 315 mothers and 22 fathers, and their children (197 females and 140 males aged 9–17 years) took part. More details regarding study recruitment, measures and sample characteristics can be found elsewhere.^15^,^18^

As part of this longitudinal study, parents and children were assessed via semistructured interviews and questionnaires at four assessment waves between April 2007 and September 2020, aimed at assessing parent and child mental health, well-being and related outcomes. The data analysed in this paper came from interviews conducted at the fourth assessment wave, conducted between 2017 and 2019, where a qualitative component was included in the interviews. This involved a series of open questions that allowed participants to share their views and researchers to use prompts and follow-up questions as needed for participants to elaborate on responses (online supplemental file 1). One of these questions aimed to explore participants’ research priorities—parents and their young adult children were asked: ‘What do you think would be important for researchers working in this area (mental health) to look into?’. Answers to this question are the focus of this study. This study is the first to report findings from this question. Transcripts of the responses to this question ranged from 2 to 795 words in length (mean word count: 109) for parents and ranged from 3 to 698 words in length (mean word count: 49) for their young adult children. 161 parents (93.3% female) aged 42–70 years and 131 young adult children (61.1% female) aged 18–28 years took part in the interviews at the fourth assessment wave. Most interviews took place in the family home with the parent and their young adult child interviewed separately by research assistants (AS, BW and JL). A small number of interviews (n=8 parents and 1 young person) were conducted remotely. More detail regarding the characteristics of the sample at the fourth assessment wave is reported elsewhere.^15^

Ethical approval was granted by the School of Medicine Ethics Committee, Cardiff University. Written informed consent was obtained for each participant.

### Analysis

Responses were analysed using QCA with an inductive approach (ie, categories were derived from the data rather than prior to data analysis), following the steps described by Elo and Kyngäs.^19^ We used QCA because it allowed a focused and clear approach to the large dataset and was best suited to achieving our research aim of identifying the participants’ research priorities. Interviews were audio-recorded, transcribed and deidentified with participants’ consent. Transcripts were coded manually by CB and double-coded independently by LO until a consensus was reached. VP, EM and CB examined the transcripts and coding framework closely to identify and agree on the categories and associated subcategories. CB and LO were undergraduate placement students working in the research department that conducted the EPAD study. VP is a postdoctoral researcher and EM is a public involvement officer; both have worked in the same research department for the past 7 years. Findings are presented grouped by category and subcategory, with illustrative quotes used to describe them, incorporating parents and their young adult children’s responses together. This is followed by a reflection on the agreement across categories between parents and young adults.

### Findings

Five categories were identified which describe the mental health research priorities reported by the parents and their young adult children. These were (1) the role of genetics in intergenerational transmission, (2) environmental and social factors, (3) treatment and intervention, (4) public understanding of mental health and (5) a developmental and intergenerational approach to research. These five categories and their associated subcategories are outlined in table 1.

**Table 1:** Overview of the categories and subcategories identified from interviews with young adults and their parents

Categories	Subcategories
(1) The role of genetics in intergenerational transmission	The ways in which mental health can be passed down through families
	The extent to which genetics contribute to mental health relative to environmental factors
(2) Environmental and social factors	Demographics
	Work and education
	Social media and the digital world
	Health behaviours
	Interpersonal relationships
	Trauma and past experiences
(3) Treatment and intervention	Improving availability, accessibility and funding
	Prevention and early intervention
(4) Public understanding of mental health	Increasing awareness
	Improving attitudes
(5) A developmental and intergenerational approach to research	Longitudinal studies of families
	Young people as the focus of research

#### Category 1: The role of genetics in intergenerational transmission

Parents and young adults in this study highlighted the role of genetics in intergenerational transmission as a priority research area. Understanding the ways in which mental health can be passed down through families (subcategory 1) was of particular importance to these participants (quote 1):

1. I think the actual genetic lab testing is good to see what’s actually going on inside someone’s body which may cause a link. (Family 1—young adult)

Some noted that understanding whether mental health is hereditary could help to inform targeted intervention (quotes 2 and 3):

2. To see whether there is any kind of link, you know, whether people are more susceptible if it does run in the family, so they can be targeted. (Family 2—young adult)

3. How maybe things that are generationally carried on. Ways of breaking the cycle. Things that can be done to ameliorate as opposed to "this is what happens". (Family 3—Parent)

The extent to which genetics contribute to mental health relative to environmental factors (subcategory 2) was raised by parents as an area of interest (quotes 4 and 5):

4. Whether the whole sort of mental health thing is a nature or nurture thing…that…would be quite interesting really whether it is a heritable thing. (Family 4—parent)

5. I suppose for me the interesting bit is how much is genetic and how much is environmental where mental health is concerned. (Family 5—parent)

#### Category 2: Environmental and social factors

Parents and their young adult children saw the influence of environmental and social factors (quote 6) and the investigation of such factors as possible causes of poor mental health (quote 7) as a research priority. Young adults and their parents noted several environmental and social factors that they felt may play an important role in mental health risk, as explored in the subcategories below.

6. Having a good awareness of people’s kind of environmental situations… and maybe the role that plays. (Family 6—young adult)

7. Different types of causes for low mood. There must be millions but it’s just knowing where it comes from would really be useful to find out. (Family 7—parent)

The influence of demographics (subcategory 1), including socioeconomic status (quotes 8 and 9), age (quote 10), gender and sexuality (quotes 11 and 12) on mental health, was highlighted as a research priority by parents and young adults:

8. Social conditions and things like that. If people are more likely to be depressed if they’re living in somewhere like [less affluent region] or living somewhere like [more affluent region]. (Family 8—parent)

9. Focus on, like, the lower end of socioeconomic sort of groups…They’re the ones who normally need the most help but they never really tend to get it. (Family 9—young adult)

10. I’d say specific age groups and the things that people have going on at different times. (Family 10—young adult)

11. I think… the relationship between gender, sexuality and mental [health]. (Family 11—young adult)

12. I think there’s really big issues with young male mental health that aren’t properly acted on…that’s where it needs to be concentrated in terms of research. (Family 12—parent)

Young adults, and their parents to a lesser extent, discussed the role of work and education (subcategory 2) in mental health as an important focus of future research, including working in an economic climate where homes are difficult to afford (quote 13), negative experiences of university (quote 14) and children’s experiences at school (quote 15):

13. A lot of people are finding it difficult to afford places as well so…How much people are working and their jobs and how that affects their mood. (Family 13—young adult)

14. I feel like people have an expectation of university being like a great place but I don’t think it is for a lot of people…I think that’s a big thing. (Family 14—young adult)

15. Maybe considering everything else that’s going on, so, for children in school, talking to their teachers so that you get a more rounded picture of what’s going on…given how many hours I suppose that they do spend at school. (Family 15—parent)

A commonly mentioned research priority, especially for young adults, was the negative influence of social media and the digital world (subcategory 3) on mental health, including mobile phone usage (quote 16) and the potential for bullying (quote 17) and peer pressure (quote 18) on social media:

16. Mobile usage…Bearing in mind that I’m at work now in front of a screen for 8 hours a day and then generally at home it will be 3 or 4 hours on my phone…I can probably see that as a direct indicator of my concentration levels and things like that. (Family 16—young adult)

17. I think with social media these days and how people can get bullied online…I think just the day and the world we live in now I think that’s probably the area to look at and keep ahead of. (Family 17—young adult)

18. Peer pressure and social media. I think there’s an awful lot of pressure put on young people today to live up to an unreasonable standard…the peer pressure that’s loaded onto the individuals has a real detrimental effect on their mental health. (Family 18—parent)

Both young adults and parents mentioned the impact of health behaviours (subcategory 4) on mental health as a research priority, including addiction (quote 19), alcohol (quote 20) and diet (quote 21):

19. They seriously need to start dealing with mental health with any kind of addiction that comes along. (Family 19—young adult)

20. I think the role of alcohol is paramount…the effect of alcoholism…on spouses and children. (Family 13—parent)

21. Diet. You know, I think that has, that has a huge impact on mental health I believe. Because if you don’t feel, I’m overweight, nothing fits, so I don’t want to go out…you then feel down, you eat more and that’s the spiral you go down. (Family 20—parent)

Looking at the role of interpersonal relationships (subcategory 5) was another suggested research priority, including familial relationships (quote 22), romantic relationships (quote 23) and loneliness (quote 24):

22. It’s all about parenting, being loved and cared for, children knowing what it’s like to be cwtched [Welsh for ‘cuddled’] and saying what they like and dislike. That’s where I see that mental health research should be invested into. (Family 21—parent)

23. Ending a relationship is a big, oh god, a big deal to all, you know, people’s depression. (Family 22—parent)

24. Probably isolation and loneliness, because, you know…There’s quite a lot of people who just don’t see people. (Family 23—young adult)

Young adults and their parents also discussed the need to investigate the role of trauma and past experiences (subcategory 6) in mental health (quotes 25 and 26):

25. Past experiences that people have gone through…and then present worries and seeing how they link. (Family 24—young adult)

26. I think the most important thing is pinning down very first trauma… it’s getting there early if there is an early trauma to deal with. (Family 25—parent)

#### Category 3: Treatment and intervention

Young adults and parents in this study highlighted treatment and intervention as a research priority. Improving availability, accessibility and funding (subcategory 1) was commonly mentioned, including the need for more treatment options and available services (quotes 27–29), support at the family level (quote 29), and the role of General Practitioners (GPs—family doctors) as the first port of call for mental healthcare in the UK (quote 30):

27. Making it easier to access help, like less waiting list for counselling…I don’t know, like it never felt that tailored…I think yeah having more, more options that are available more easily. (Family 26—young adult)

28. Rather than people just like being put on medication like other root ways I think may be solving the problem more… I don’t know I just feel like doctors are very quick- they just like stick you on whatever [medication] and that’s not really what you want, even though it does help. (Family 27—young adult)

29. More needs to be done in the way of the mental health systems in this country. There needs to be much more investment in it, there needs to be many more psychiatrists, psychotherapists, um… more frequent appointments… much more advice and help to the families of people suffering from mental health issues. (Family 28—parent)

30. The way that our GP system works is really important because that’s your first port of call… I think it tends to exacerbate things the way it is to actually get in touch with the GP… and if you are feeling stressed it exacerbates that… Once you get through that part to a diagnosis then things tend to get slightly easier. (Family 29—parent)

Parents and their young adult children noted prevention and early intervention (subcategory 2) as a research priority, including the need for early detection and diagnosis (quotes 31–33):

31. Early diagnosis. People who don’t understand their own low mood and depression and who don’t seek [help]…it’s left too late, because earlier intervention would obviously work a lot better. (Family 30—parent)

32. Trying to prevent them [mental health disorders], sort of before medication and things. (Family 31—young adult)

33. An early scoring system would be really good, um, especially…with anxiety and depression. (Family 17—parent)

#### Category 4: Public understanding of mental health

Public understanding of mental health was highlighted as a research priority by parents and young adults, including increasing awareness (subcategory 1) to improve understanding of mental health issues in general (quote 34), as well as in schools (quote 35), workplaces (quote 36) and families (quote 37):

34. I think the campaign of awareness should carry on because it’s definitely beneficial. (Family 32—parent)

35. I think it’s got such a stigma, and so many people experience it without realising. I think it’s probably a good idea to raise more awareness in schools. (Family 33—young adult)

36. There needs to be more understood about mental health…It’s a huge problem in the workplace. (Family 19—parent)

37. If it helps families to understand what’s going on with each other, you know, when one member of the family has got depression, it’s got to be a good thing. (Family 34—Parent)

Parents and their young adult children also discussed the importance to them of research focused on improving attitudes (subcategory 2) surrounding mental health, including fostering acceptance and ‘breaking the taboo’ (quotes 38–40):

38. People’s attitudes are much better now…I think than many, many years ago…but I still think there needs to be a lot more acceptance of it, in that people who do suffer with mental health, they can’t help it. (Family 35—parent)

39. I still think there a big issue of mental health and how it’s a taboo subject which needs to open up even more than it has done already. (Family 36—parent)

40. I think [research] is important because people don’t talk about (mental health) enough…Making it…a more talked about thing and that people are okay with going to see people about certain stuff. (Family 37—young adult)

#### Category 5: A developmental and intergenerational approach to research

Longitudinal studies of families (subcategory 1) affected by mental health difficulties were seen as a research priority. The value of taking a developmental approach, similar to the EPAD study that participants had taken part in, was noted (quotes 41 and 42):

41. It’s just interesting seeing, over the years then, like since I last spoke to you and stuff, like the outcomes of…have I stayed in a job, education, like stuck at things and, and have you got any mental health problems now. (Family 38—young adult)

42. It’s really valuable to have such a longitudinal study…So uh more of the same, more of the longitudinal stuff, because you really get a feel for how families develop. (Family 39—parent)

For young adults, young people as the focus of research (subcategory 2) was an important consideration (quotes 43 and 44):

43. You need to look at kids, children. (Family 40—young adult)

44. More focus on…child mental health. (Family 29—young adult)

#### Agreement of data from parents and young adults

All five categories identified were supported by data from both the parents and their young adult children. However, some categories were more commonly discussed as research priorities by parents compared with young adults and vice versa. ‘Treatment and intervention’ was the most commonly suggested focus for research, although this category was more commonly discussed by parents than young adults. The categories of ‘the role of genetics in intergenerational transmission’ and ‘public understanding of mental health’ were also mentioned as research priorities more by parents. The impact of ‘environmental and social factors’ on mental health was mentioned as a research priority more by young adults. Finally, while both parents and their young adult children talked about the value of ‘a developmental and intergenerational approach to research’, only young adults explicitly mentioned the subcategory ‘young people as the focus of research’ as a priority.

## Conclusions

We aimed to explore the mental health research priorities of parents with a history of depression and their young adult children. In line with previous work involving members of the general public,^4^,^7^ young adults and parents in this study highlighted treatment and intervention, including prevention and early intervention, as research priorities. Public understanding of mental health was also highlighted as a research priority, as well as environmental or social factors that might contribute to poor mental health. In terms of how well these identified priorities map onto mental health research being conducted in the UK, according to a report by MQ of UK mental health research funding between 2014 and 2017, research into factors contributing to poor mental health received the most funding, while treatment development and prevention were two of the most poorly funded areas.^20^ However, in the UK, there have been recent funding investments into treatment and intervention development, as illustrated by the National Institute for Health and Care Research annual report^21^ and strategic funding calls in this area.^22^ While funding for research into public understanding of mental health was not reported in the MQ report, stigma is one aspect of public understanding and a review of stigma research in families with parental mental illness highlighted this as an area with relatively little research.^23^ Our findings suggest that research into public understanding of mental health is an important priority for parents with depression and their children.

The role of genetics in intergenerational transmission being highlighted as a priority for mental health research seems to be a novel finding and not one that has been reported in priority-setting exercises involving the general population.^4^,^7^ In this study, young adults and their parents were interested in the way in which mental illness can be passed down through families and the extent to which genetics contribute to mental health relative to environmental factors. Recent youth mental health funding, at least in the UK, has had an increased focus on intervention development, with calls to focus more attention on this and less on research into neurobiological mechanisms of mental ill health.^24^ However, our study indicates that for families affected by common mental health problems, fundamental research about aetiology, including genetically informed research, is also viewed as an important priority. Literature on psychiatric genetic counselling highlights that families affected by mental illness often overestimate the genetic risk to children,^25^ despite evidence from genetically informed studies demonstrating a comparable contribution of environmental influences to genetic influences in the intergenerational transmission of mental health.^26^ The research priorities identified in this study likely reflect the experiences of this group of parents and children, and some categories may be interrelated (eg, intergenerational transmission and public understanding of depression). For instance, knowledge of the relative contribution of genetic and environmental factors to mental health, and how this is communicated to patients, is likely to play an important role in an individual’s understanding of their own condition and potential feelings of self-stigma.^25^ These findings highlight the importance of effectively communicating current research knowledge to patients and the public in areas of importance to them, including knowledge that could help inform individuals about the relative role of genetics in the familial transmission of mental health. Knowledge exchange activities between researchers and the public, in addition to awareness-raising activities of bodies such as third-sector organisations, may help to break down barriers between research, policy and practice.

Findings should be considered in light of the study’s strengths and limitations. While several fathers contributed to the data analysed in this study, the parents who participated were mostly mothers, and therefore the research priorities found may not generalise to fathers. We report findings from a small qualitative component within a longer interview aimed at assessing parent and child mental health, well-being and related outcomes in a large, prospective, longitudinal cohort study. Therefore, the recruitment of participants and sample size were based on the requirements for the longitudinal cohort study and not with qualitative study sample size saturation in mind, and thus the number of participants exceeds what one would typically expect to see in a qualitative study. Participation in a longitudinal study of parents with a history of depression and their children may also impact upon some of the views expressed by parents and young adults, for example, their positive views on longitudinal follow-up of families affected by mental health problems and their interest in the way in which mental health can run in families. However, this study provides a unique example of integrating qualitative data collection into a longitudinal cohort study, providing a novel and cost-effective opportunity for in-depth exploration of the research priorities of a large number of families affected by depression. Integrating qualitative semistructured questions allowed us to understand research priorities shaped and initiated by the participants themselves, rather than being constrained to the pre-empted responses of a questionnaire, for example. As the qualitative questions were part of a larger, more structured interview, there was less opportunity to gather wider contextual insights on each participant and their experience. Therefore, QCA, rather than other qualitative analytic approaches such as thematic analysis, was considered the best-suited and efficient way to achieve our research objective—to produce a list of overarching categories for future research from the perspective of parents and their young adult children. QCA was also well suited for analysing the large dataset in a systematic manner with multiple data analysts, enhancing the rigour of our research.

### Clinical implications

These findings have implications for future mental health research directions and highlight the value of including the unique perspectives of diverse groups, including those with lived experience of mental health, in priority-setting exercises. The priorities identified can help to inform the decisions of funding bodies that increasingly recognise the importance of consulting the research priorities of stakeholders when setting research funding agendas.^12^ Families affected by depression viewed research questions investigating the relative role of genetic and environmental factors, treatment and intervention, and public understanding of mental health as important priorities, in addition to the importance of developmental and intergenerational research, and parents and their young adult children shared many common views.

## Supplementary material

10.1136/bmjment-2024-301279 online supplemental file 1

## Data Availability

No data are available.
