# Supplementary material for: Exploring the mental health research priorities of parents with depression and their children
Source: BMJ Ment Health. 2025 Jun 22;28(1):e301279. doi: 10.1136/bmjment-2024-301279 (PMC12184385; doi:10.1136/bmjment-2024-301279)

**Supplement 1: Open questions for parents and young adults (interviewed separately)**

*Note: Answers to the fourth question only were analysed in this study*


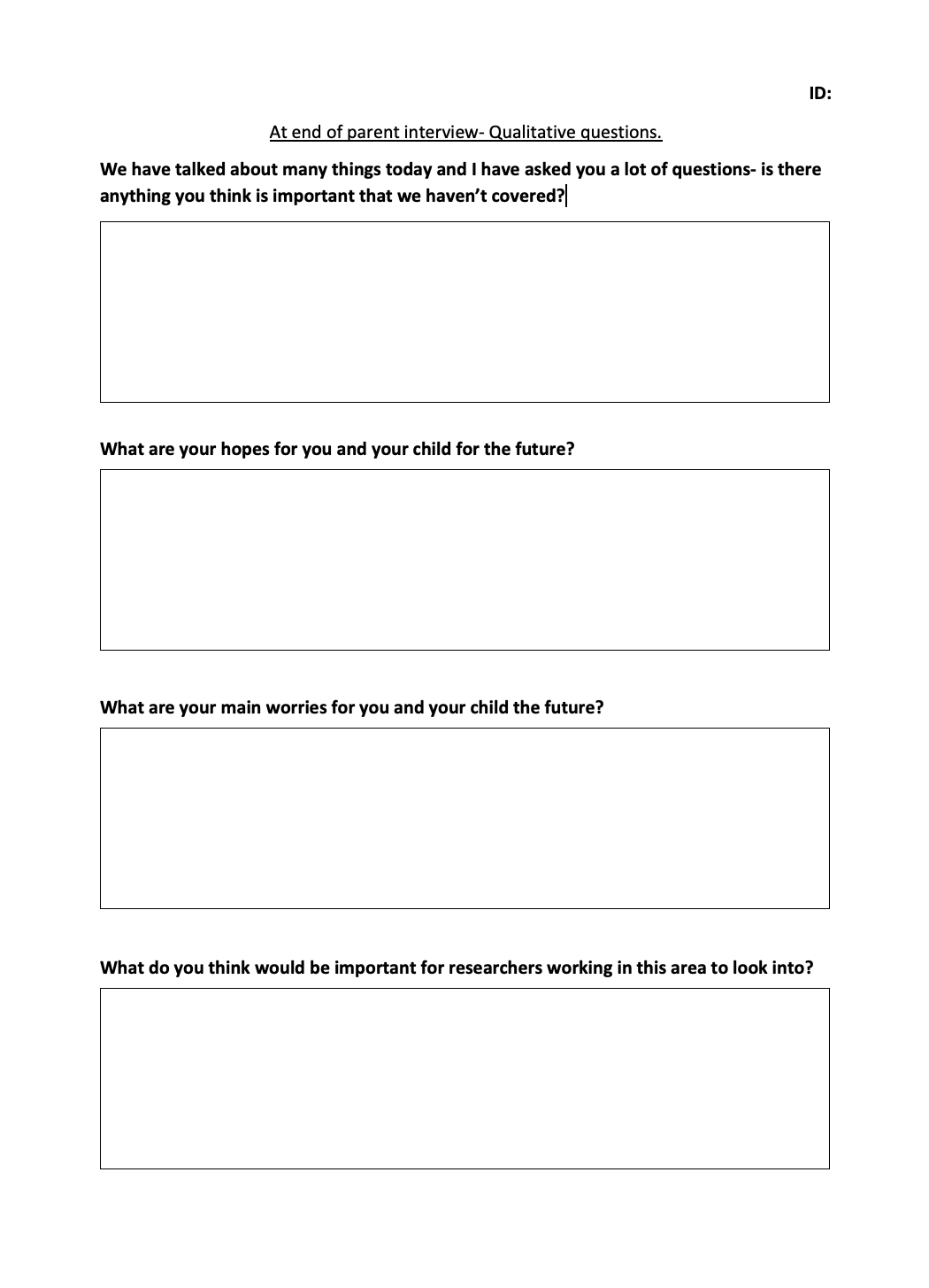

Supplement: online supplemental file 1 [file bmjment-28-1-s001.docx]
